# Wavelet-Based Filtration Procedure for Denoising the Predicted CO_2_ Waveforms in Smart Home within the Internet of Things

**DOI:** 10.3390/s20030620

**Published:** 2020-01-22

**Authors:** Jan Vanus, Klara Fiedorova, Jan Kubicek, Ojan Majidzadeh Gorjani, Martin Augustynek

**Affiliations:** Department of Cybernetics and Biomedical Engineering, VSB-Technical University of Ostrava, FEECS, 708 Ostrava-Poruba, Czech Republic; klara.fiedorova@vsb.cz (K.F.); jan.kubicek@vsb.cz (J.K.); ojan.majidzadeh.gorjani@vsb.cz (O.M.G.); martin.augustynek@vsb.cz (M.A.)

**Keywords:** intelligent buildings, wavelet transformation, prediction, artificial neural network, multilayer perceptron, cloud computing, Internet of Things, smart home

## Abstract

The operating cost minimization of smart homes can be achieved with the optimization of the management of the building’s technical functions by determination of the current occupancy status of the individual monitored spaces of a smart home. To respect the privacy of the smart home residents, indirect methods (without using cameras and microphones) are possible for occupancy recognition of space in smart homes. This article describes a newly proposed indirect method to increase the accuracy of the occupancy recognition of monitored spaces of smart homes. The proposed procedure uses the prediction of the course of CO_2_ concentration from operationally measured quantities (temperature indoor and relative humidity indoor) using artificial neural networks with a multilayer perceptron algorithm. The mathematical wavelet transformation method is used for additive noise canceling from the predicted course of the CO_2_ concentration signal with an objective increase accuracy of the prediction. The calculated accuracy of CO_2_ concentration waveform prediction in the additive noise-canceling application was higher than 98% in selected experiments.

## 1. Introduction

In the field of intelligent building (IB) automation and in the context of optimized management of operational-technical functions to reduce operating costs, increase control, and comfort, the European Union has published the directive “Directive (European Union) 2018/844 of the European Parliament and of the Council of 30 May 2018” emphasizes monitoring and processing of measured data in real time. Building automation and electronic monitoring of building technical systems offer considerable potential for cost-effective and significant energy savings for both the consumers and businesses [1].

In this article, the authors describe the implementation of KNX (Konnex (standard EN 50090, ISO/IEC 14543) technology with IBM Internet of Things (IoT) platform connectivity for monitoring and processing of measured data in real time within IB automation. On the basis of the measured values of carbon dioxide (CO_2_) concentration, it is possible to detect the occupancy of the monitored smart home (SH) spaces, the arrival of a person into the monitored room, or the exit from the monitored space, or the length of stay in the monitored space. The aforementioned procedure enables the unambiguous determination of the occupancy rate of the monitored SH spaces, indirectly by measuring common nonelectrical quantities of CO_2_ within the operational–technical function control in the SH. In market research, we ascertained that CO_2_ sensors are two to three times (in some cases, greater) more expensive than temperature and humidity sensors, which are, moreover, a common part of IB in the Czech Republic.

Due to the higher costs of acquiring CO_2_ sensors, we proposed the possibility of lowering the initial investment costs for IB by providing information about the occupancy of the individual rooms within a novel indirect method for monitoring IB spaces presence occupancy using a temperature indoor sensor and relative humidity sensor instead of a CO_2_ indoor sensor. This method uses predictive modeling (using Statistical Package for the Social Sciences from the company IBM (SPSS) Modeler 18) with the application of an artificial neural network (ANN) for the prediction of CO_2_ concentration using the measured values of indoor temperature and indoor relative humidity. To increase the accuracy of this method, the additive noise canceling method was used with wavelet transformation. This article emphasizes the adjustment and optimization of individual parameters of wavelet transformation (mathematical filtration method) for additive noise canceling and an increase in CO_2_ prediction accuracy. Additionally, this article illustrates the data collection and the IoT platform connectivity within KNX technology as a practical part of (SH) control simulation. The main goal of the authors is to find the optimal setting of individual parameters of the wavelet transform in the additive noise suppression application with an emphasis on increasing the accuracy of predictions of CO_2_ concentration from measured values of indoor temperature and indoor relative humidity for monitoring and recognition of IB space occupancy using common operation sensors.

### Related Works

The monitoring of technical systems can be utilized using android mobile visualization applications [2] (the prediction was performed by the ANN Bayesian regulation method (BRM) with least mean square (LMS) adaptive filtration (AF) additive noise canceling, best accuracy was better than 90%), supervisory control and data acquisition (SCADA) visualization systems, or robust software (SW) tools for collecting and archiving measured data in smart home care (SHC) [3] (the prediction was performed by the ANN-based on the Levenberg–Marquardt algorithm (LMA), experimental results verified high method accuracy > 95%). Similarly, the IoT platform can also be employed to monitor and visualize technical systems in IB [4]. KNX is one of the many technologies that are widely used to control the technical and operational functions of IB worldwide [5]. Petnik et al. describes the implementation of KNX technology for controlling and monitoring operational and technical functions in SHs within the IoT cloud platform [6]. The SHC platform and health care platform [7,8] are being prepared to use the IoT concepts within the fifth generation of the mobile network standard [9].

Measured values of nonelectrical and electrical quantities in real time using implemented KNX technology in SH (presence of persons, power consumption, temperature, relative humidity, or CO_2_ concentration) need to be preprocessed and adjusted for subsequent calculations using appropriate mathematical methods (classification [10,11], recognition [12,13,14], and prediction [15]) (the prediction was performed by the ANN-based on the scaled conjugate gradient (SCG), experimental results verified high method accuracy > 90%), [16] (the prediction was performed by the ANN Bayesian regulation method (BRM) with LMS AF additive noise canceling, best accuracy was better than 95%) [17] (the prediction was performed by decision tree regression method with the accuracy of 46.25 ppm). An important area of the described chain is the suppression of additive noise from the measured and calculated waveforms of monitored quantities [18,19]. The disadvantage of using an LMS adaptive filter in an additive noise suppression application is the slow startup of the filtering process in the initial phase, depending on the step size parameter set and the adaptive filter order.

Therefore, we decided to use the wavelet transform in an application to suppress additive noise from the predicted CO_2_ signal in order to increase the accuracy of CO_2_ prediction.

The signal noise represents a significant problem for signal representation and further processing [20]. Each signal is represented by the trend component, determining the signal evolution over time [21]. The further signal components, including periodic and aperiodic parts, are superimposed on the signal trend [22]. These components standard represent an additive part having the noise character, thus, deteriorate the visual quality and features of the CO_2_ signals [23]. Therefore, we aimed to extract the trend component of the CO_2_ signal, while the additive noise is supposed to be suppressed. For noise suppression, many methods have been developed. Mostly, these methods utilize a sliding window, passing through the signal area and approximate local features of the signals by statistical parameters, such as the average or median filters, adaptive filters, or fitting procedures, including the Savitzky–Golay filter [24,25]. Nevertheless, such methods are not capable of adjusting to the local frequency content, except for the adaptive filters which are time-consuming and require a reference signal, which can be a complication. An approximation of the local frequencies by an adjustable window function is crucial for the nonstationary signals, where we observe time-varying frequency content over time [26,27,28]. For this reason, we apply the wavelet-based filtration with the goal of time-frequency localization of the CO_2_ signal trend [29,30,31].

This study is divided into the following sections: The Introduction provides the motivation and current state of the art on the topic of wavelet-based filtration procedure for denoising the predicted CO_2_ waveforms in SH within IoT. The next section describes building automation and data collection with KNX technology, preprocessing of the collected data, predictive modeling (using IBM SPSS Modeler 18), additive noise cancelation with wavelet transformation, description of experiments, and evaluation of the obtained results. Finally, the results are discussed with comparisons to existing solutions.

## 2. Materials and Methods

**Figure 1:**
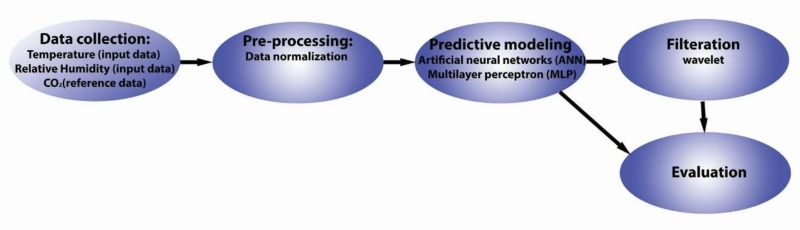
Block diagram of the analysis.

The practical implementation of prediction and filtration of CO_2_ waveforms are divided into the following parts (Figure 1):

1. Building automation and data collection (using KNX technology);
2. Preprocessing of the collected data;
3. Predictive modeling (using IBM SPSS Modeler 18);
4. Filtration (wavelet transformation);
5. Evaluation of the obtained results.

### 2.1. Building Automation and Data Collection Using KNX Technology

KNX is a worldwide standard (EN 50090, ISO/IEC 14543) for building automation. The creators and owners of KNX technology are the KNX Association. Product certification based on the KNX standard guarantees the compatibility of products of different companies (Siemens, ABB, Schneider Electric, WAGO, and others), which represents a high level of flexibility. KNX technology is a decentralized system, i.e., the KNX bus system that does not need a PC or a central control unit to operate. All of the information and data are stored in microprocessors of the individual KNX modules (KNX bus participants) which communicate with each other on the same level, the so-called multi-master communication. Commissioning is done using the Engineering Tool Software (ETS) software. KNX can provide a variety of applications for lighting control, sun protection, heating, cooling, ventilation, energy management, convenience control, etc. The KNX application is used to control the operational-technical functions of office buildings, shopping centers, medical facilities, institutions, banks, industrial locations, etc. This control system does not only bring the comfort of operation but above all, it is an efficient tool for efficient control of operational technical functions.

**Figure 2:**
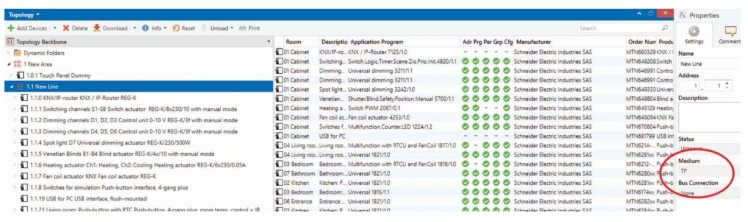
Created KNX topology and selected medium TP KNX in the software (SW) tool ETS 5.

To simulate SH operation, KNX test panels (containing KNX modules) were placed in the laboratory EB312 at the new FEI (Faculty of Electrical Engineering and Computer Science) building at the VSB Technical University of Ostrava. This location often holds educational classes, or it is visited by staff and researchers. Using the modules displayed in Figure 2, it was possible to simulate the control of operational functions in SH. The measurements of CO_2_ accumulation, indoor temperature, and humidity were performed using the MTN6005-0001 module. The measuring range of this device is listed below:

- CO_2_ sensor, 300 to 9999 ppm;
- Temperature sensor, 0 °C to +40 °C;
- Air humidity sensor, 20% to 100%.

In KNX topology, there are the following four available communication media for the actual transmission of data telegrams between individual KNX modules: Twisted Pair (TP) (also known as KNX bus), powerline (PL), radio frequency (RF), and ethernet (IP). Each communication medium can be used in combination with one or more configuration modes. Economical operation and comfort in the control of operational-technical functions are the main priorities of implementations within family houses. Therefore, TP was selected as the backbone structure of this implementation (Figure 2).

**Figure 3:**
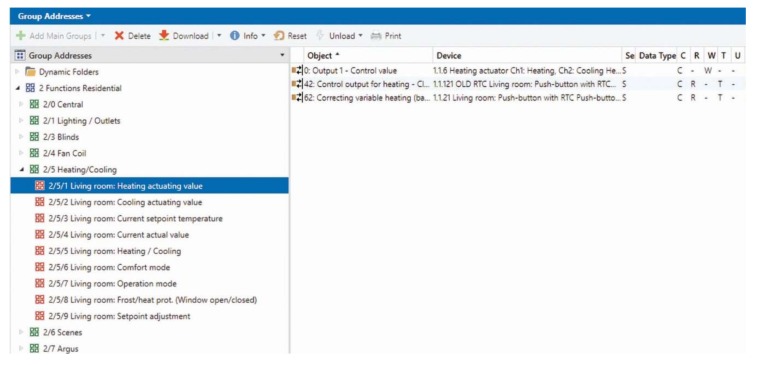
Group address structure created to simulate the management of operational and technical functions in the Engineering Tool Software (ETS) tool.

The operation of the individual components of KNX technology is ensured by the means of group addresses (Figure 3).

**Figure 4:**
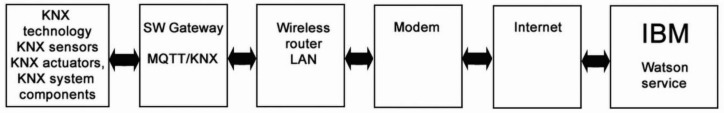
Block diagram of created SW connection between KNX technology on the building of new FEI VSB-TU Ostrava and IBM IoT Watson platform [29].

The connection of KNX technology and IBM cloud technology is ensured in this work by our developed software [32], which enables the communication between IBM Watson IoT platform and KNX smart installation (Figure 4). Message queuing telemetry transport (MQTT) protocol is used as a communication protocol.

### 2.2. Preprocessing of the Collected Data

Data normalization (using feature scaling) was selected as the preprocessing stage. Feature scaling (min–max normalization) is rescaling the range of features to the scale of zero to one. Mainly, the feature scaling is applied because the gradient descent converges much faster with feature scaling than without it. The general formula for a min–max is given as [29]:

(1) $$\mathrm{Normalized}\;\mathrm{value}\;=\;\frac{\mathrm{current}\;\mathrm{value}-\mathrm{minimum}\;\mathrm{value}}{\mathrm{maximum}\;\mathrm{value}-\mathrm{minimum}\;\mathrm{value}}$$

### 2.3. Predictive Modeling (Using IBM SPSS)

Predictive models are based on variables (predictors) that are most likely to influence the outcome (prediction) [33]. This article employs a predictive model that is categorized as machine learning with a supervised learning strategy. Machine learning can be described as the process of computers making intelligent decisions by learning and recognizing patterns based on the sample data. In a supervised learning strategy, the machine establishes a pattern between the problem and the answer by learning from a set of solved (labeled) examples. Once the pattern is established the machine is able to solve similar problems [34]. ANNs are one of the most popular modeling methods used in predictive applications (such as [35,36,37,38,39,40,41,42]), due to their power flexibility and ease of use. In general, ANNs obtain their knowledge from the learning process and then use interneuron connection strengths (known as synaptic weights) to store the obtained knowledge [43,44]. One of the most commonly used classes of ANNs is a multilayer perceptron (MLP). MLP is a feedforward neural network. In addition, input and output layers of the MLP can contain multiple hidden layers (at least one) and each can contain multiple neurons. The MLP utilizes backpropagation for training [45,46,47]. Due to its multiple layers and nonlinear activation, MLP can distinguish data that is not linearly separable [48].

**Figure 5:**
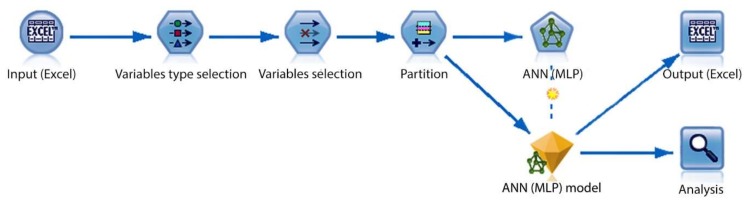
Developed data stream in IBM SPSS modeler.

The MLP ANN was implemented in the IBM SPSS Modeler 18 software tool. Figure 5 displays the developed data stream. Initially, the input data was imported to the data stream (using Excel node). The filter and type were utilized to select relevant input data, assign correct variable types (continuous, categorical, etc.), and predefining inputs and the outputs. The data stream continues with a partitioning node with a predefined ratio of 40% training, 30% testing, and 30% validation. Commonly, K-fold, V-fold, N-fold, and partitioning methods are used to evaluate the performance of the developed models in the IBM SPSS modeler. K-fold, V-fold, and N-fold are splitting methods that divide the dataset into as many parts as there are possible values for a split field. Splitting results in every input vector are used for training and validation (by building multiple models). Unlike splitting, partitioning is used to evaluate the performance of a single model. It randomly divides the input dataset into three parts of training, testing, and validation. It provides a good indication of model performance by using one sample to generate the model and a separate sample to test and evaluate it. In general, partitioning is an optimal validation method for building a single model with the large datasets. Using validation partition, the built models can perform predictions using only predictors. In the next stage, the partitioned data are fed into the ANN modeling node, and the IBM SPSS modeler algorithm guide [49] mathematically describes its MLP model as followings:

**Input layer**$j_{0\;}=p$ units, $a_{0:j},\ldots ,a_{0:j0}$, with $a_{0:j}=xj$, where j is the number of neurons in the layer and X is the input.

***i*th hidden layer**$j_{i}$ units, $a_{i:1},\ldots ,a_{i:j_{i}}$, with $a_{1:k}=\;\gamma_{i}\left(C_{i:k}\right)$ and $C_{i:k}=\sum_{j=0}^{j_{i-1}}\omega_{I:j1},{\;k}^{a_{i-1:j}}$, where $a_{i-1:0}=1$, $\gamma_{i}$ is the activation function for the layer I, and $\omega_{I:j1}$ is weight leading from layer i−1. At this layer the model uses hyperbolic tangent as an activation functions given by $\gamma_{\left(C\right)}=\tan h\left(c\right)\frac{e^{c}-e^{-c}}{e^{c}+e{-}^{c}}$.

**Output layer**$j_{I}=R$ units, $a_{I:1},\ldots ,a_{I:J_{I}}$, with $a_{I:k}=\;\gamma_{I}\left(C_{I:k}\right)$ and $C_{I:k}=\sum_{J=0}^{J_{1}}\omega_{I:j},k^{a_{i-1:j}}$, where $a_{i-1:0}=1$. For continuous prediction signal at this layer, the model uses identity ($\gamma_{\left(C\right)}=c$) as an activation function.

Training or estimation of the weights is divided into the following three stages:

- Initialization of the weights (using alternated simulated annealing and training procedure);
- Computing the derivative of the error function with respect to the weights (via the error backpropagation algorithm);
- Updating the estimated weights (via gradient descent method).

The resulting model (displayed as nugget gem) can export its predictions to Excel files (using excel node) or analyze them using built-in functions such as plots and analysis nodes.

### 2.4. Wavelet Filtration

In the wavelet transformation, we operate with variable complex window functions, which are assigned into wavelet families. These groups of the wavelet functions differ from each other by the frequency features and morphological structure for the extraction of the specific features from the noisy signals. In this context, a selection of a suitable wavelet function is essential for the proper trend detection. Furthermore, it is important to mention that the wavelet transformation allows for the CO_2_ signal decomposition into individual decomposition levels, keeping certain trends and detail information, while the rest is irreversibly suppressed [50,51]. On the basis of this procedure, we can build the wavelet-based filter bank, allowing for the CO_2_ signal decomposition in multiple levels. In this context, it is crucial to select an appropriate decomposition level to detect the CO_2_ signal trend and simultaneously suppress the signal details, representing the image noise, or improper prediction of the ANNs. Another aspect of wavelet-based filtering is the settings of filtration. Wavelet-based filtration is based on the fact that some approximation and detail coefficients from the wavelet transformation represent the signal noise, other than the signal trend. Those which contain the signal noise are suppressed by applying the thresholding procedure, where we need to select a suitable threshold and thresholding. These aspects of the wavelet transformation, including the type of the wavelet, level of decomposition, and thresholding rules, are input parameters on the basis of which we build the filtration procedure for the wavelet-based smoothing of CO_2_ signals. In this paper, we present a comparative analysis of different settings of the wavelet analysis to achieve the most suitable procedure for the CO_2_ signal smoothing with the aim of improving the accuracy of ANN prediction. All the settings are objectively verified based on selected evaluation parameters against the reference CO_2_ signals [52,53,54].

#### Concept of Wavelet Transformation

As we stated before, the CO_2_ signal standard contains time-varying frequency content, which makes this signal nonstationary. Therefore, we can use the time-frequency localization via using the window function, called wavelets. In this context, it is important to note that this window function is related to varying resolution in the time and frequency domain. According to the principle of uncertainty, the longer the window is, the better the frequency resolution we achieve and vice versa.

This predetermines the fact that it is impossible to achieve a perfect simultaneous localization of the low- and high-frequency components by using the window function with a constant length. In this context, on the one hand, the discrete wavelet transformation (DWT) enables the following benefits: The dynamic window function enables optimization of the time-frequency localization of different scales, enables the CO_2_ signal decomposition in different levels with different level of the details and trend suppression, and enables use of the complex window function in the comparison with elementary window functions which are used in the short-time Fourier transformation (STFT). On the other hand, we need to consider the limitations of the wavelet transformation. Mainly, it is plenty of settings, including the mother’s wavelets, level of decomposition, and the thresholding rules. These parameters are different for individual applications, and there is not a versatile way to determine the best setting for a particular task [55,56]. The definition of the DWT is given as follows:

(2) $$d_{j,n}=\langle x\left(t\right),\;\psi_{j,n}\left(t\right)\rangle =\int_{-\infty }^{\infty }x\left(t\right)\;\bar{\psi_{j,n}\left(t\right)}dt\;,$$

where $\psi_{j,n}\left(t\right)$ represents the mother wavelet with the following definition:

(3) $$\psi_{j,n}\left(t\right)=a_{0}^{-\frac{j}{2}}\psi \left(a_{0}^{-j}t-nb_{0}\right).$$

In this definition, the parameter $a_{0}$ stands for the scaling and $b_{0}$ is translation. These parameters are selected so that $\psi_{j,n}\left(t\right)$ has the orthogonal bases. Using $a_{0}=2$ and $b_{0}=1$ we obtain the equation for the mother wavelet as follows:

(4) $$\psi_{j,n}\left(t\right)=2^{-\frac{j}{2}}\psi \left(2^{-j}t-n\right).$$

Using Equation (2), the orthonormal wavelet transformation is given as follows:

(5) $$d_{j,n}=\langle x\left(t\right),\;\psi_{j,n}\left(t\right)\rangle =2^{-\frac{j}{2}}\int_{-\infty }^{\infty }x\left(t\right)\;\bar{\psi_{j,n}\left(2^{-j}t-n\right)}\;dt.$$

The inverse transformation is given by:

(6) $$x\left(t\right)=\sum_{j=1}^{J}\sum_{n=1}^{N}d_{j,n}\psi_{j,n}\left(t\right).$$

The discrete wavelet transform is computed in the consecutive steps by applying low-pass and high-pass filters for the definition of the approximation and detail coefficients for each level of decomposition. This decomposition scheme represents a binary tree in the form of the Mallat algorithm with the goal of the multiresolution analysis as a filter bank. At each decomposition level, these half-band filters pass the signal with a half frequency band. This decimation by two down-sampling halves the time resolution and the signal is represented by half of the original samples. By using this approach, we achieve arbitrarily optimal time resolution in high frequencies and the frequency resolution in low frequencies. The process of decomposition is repeated until the maximal level of the decomposition is reached. This level is depended on the signal length. The inverse reconstruction of the original signal is done via sequences of the approximation and detail coefficients begins at the last decomposition level [57,58]. The signal inverse reconstruction undergoes all the levels of the decomposition.

### 2.5. Evaluation Methods

**Accuracy** The accuracy of the built models was obtained using the following expression:

(7) $$\mathrm{Accuracy}\;=\;\frac{1}{n}\sum_{m=1}^{M}(1-\frac{\left|y_{i}{}^{\left(m\right)}-\right.\left.{\hat{y}}_{i}{}^{\left(m\right)}\right|}{max_{m}\left(y_{i}{}^{\left(m\right)}\right)-min_{m}\left(y_{i}{}^{\left(m\right)}\right)}).$$

**Mean Square Error (MSE)** This measures the average of the error squares between two signals. It is given by the following mathematical expression [59]:

(8) $$MSE=\;\frac{1}{n}\sum_{i=1}^{n}{\left(y_{i}-{\hat{y}}_{i}\right)}^{2}.$$

**Linear Correlation (LC)** This corresponds to a degree of dependence (correlation) between two variables. It is given by the mathematical description [60]:

(9) $$\mathrm{Linear}\;\mathrm{Correlation}=\frac{n\sum_{i=1}^{n}y_{i}{\hat{y}}_{i}-\sum_{i=1}^{n}y_{i}\sum_{i=1}^{n}{\hat{y}}_{i}}{\sqrt{n\sum_{i=1}^{n}y_{i}^{2}-{\left(\sum_{i=1}^{n}y_{i}\right)}^{2}}\;\sqrt{n\sum_{i=1}^{n}{\hat{y}}_{i}^{2}-{\left(\sum_{i=1}^{n}{\hat{y}}_{i}\right)}^{2}}}.$$

## 3. Results

### 3.1. Data Collection

The measurements were performed in the laboratory EB312 on the premises of the new FEI building of the VSB Technical University of Ostrava. The data collection started on May 2 at 10:08:06 and ended on May 10 at 11:52:45 (a weeklong data interval). Using the developed software, the data collection rate can vary between one to ten samples per minute. Resulting in a total of 55,241 samples. This location often holds educational classes, or it is visited by staff and researchers. However, during the days 4th (Saturday), 5th (Sunday), and 8th (public holiday “Victory in Europe Day”) of May the measurement room (laboratory EB312 in new FEI building of VSB Technical University of Ostrava) remained unoccupied.

### 3.2. Predictions and Evaluations

Table 1 shows the obtained result from evaluating the validation partition with respect to the reference signal. The accuracy, MSE, and LC coefficient were used for objective evaluation of the developed models. The lowest accuracy was obtained by Model Number 7 (accuracy, 91.6%; LC, 0.956; and MSE, 2.525 × 10^−3^) and Model Number 3 resulted in the highest prediction accuracy (accuracy, 96.7%; LC, 0.983; and MSE, 9.78 × 10^−4^). It can be observed (Table 1) that most of the obtained models result in similar accuracies. Therefore, a number of neurons do not significantly impact prediction accuracy. The complete and detailed analysis of these prediction results can be found in [32].

### 3.3. Filtration and Evaluation

#### 3.3.1. Wavelet Settings for CO_2_ Signal Prediction

In this section, we describe the wavelet transformation settings for the CO_2_ signals smoothing. The predicted signals mostly contain additive signal noise, exhibiting steep fluctuations that have a nature to deteriorate the CO_2_ signal trend. Therefore, we aimed to eliminate the signal details which do not have the origin of the ambient CO_2_ concentration, but are the product of the ANN, depending on the number of the neurons in the ANN.

In our analysis, we use the one-dimensional (1D) model of the wavelet transformation, which is a one-dimensional function, serving for the noise suppression. Wavelet transformation transforms the original CO_2_ signal samples on the sequence of the wavelet coefficients. The wavelet-based filtration is consequently based on the thresholding of the wavelet coefficients. An essential part of the analysis is selecting the suitable settings of the wavelet filtration with the goal of optimal extraction of the CO_2_ signal trend part while suppressing other details. Before applying the wavelet filtration, we tested and adjusted wavelet filter parameters. On the basis of the testing, we used the following settings for the signal smoothing (Table 2).

In this work, we use filtering based on adaptive threshold selection using the principle of Stein’s unilateral risk estimate (SURE), which is called rigrsure. A threshold is for the soft threshold estimator. Starting with an estimate of risk for a particular threshold value, t, the algorithm minimizes the risks to yield a threshold value. For the soft thresholding, values for both positive and negative coefficients are "shrinked" towards zero.

Another crucial part of the analysis deals with optimized settings of the mother’s wavelets which appear as the most suitable for the analysis since individual families of the wavelets differ among each other by their morphological features, allowing for the extraction of specific signal features. In this context, it is supposed that unappropriated wavelet selections would lead to a bad CO_2_ signal approximation, and therefore an unsuitable prediction. On the basis of the experimental testing, we selected three, the most significant wavelets, a well approximating CO_2_ signal trend (Table 3).

On the basis of the experimental testing, we found the wavelet scaling to be one of the most significant parameters which significantly influences the resulting prediction. Within the wavelet smoothing, the CO_2_ signal is decomposed into a finite number of levels which gradually suppress the details of the CO_2_ signal. This task controls the ANN prediction. After experimental testing, we decided to use the following levels of the decomposition: n = 3, 6, and 10. These levels are, consequently, used for the building of the prediction model.

Since we use different settings of the wavelet transformation and different topology of the ANN, we need to objectively evaluate the efficiency and robustness of each setting to report the configuration for the CO_2_ signal prediction. For the purpose of this objective evaluation, we use MSE and correlation coefficient for objective testing. The MSE represents an error function calculated between the native predicted signal from ANN $Y\left(i\right)$ and smooth signal $\hat{\;Y}\left(i\right)$ from the wavelet-based filtration.

The next evaluation parameter is the correlation coefficient which measures a level of the linear dependency between native predicted signal and the wavelet smooth signal. In difference with MSE, the correlation coefficient is a normed parameter in the range: $\left[0;1\right]$, where zero stands for no correlation, whereas one stand for the full correlation.

#### 3.3.2. Optimization of Wavelet Settings and Testing

In this section, we present testing and optimization of the wavelet settings from the view of wavelet filtration settings and levels of the decomposition, as well as for the different time ranges of the native CO_2_ signals and selection of the ANN. In our analysis, we bring a comparative analysis of the testing trend-level (n) of the wavelet-based CO_2_ concentration predicted waveform decomposition where n = 3, 6, and 10. Each such level performs the signal decomposition into approximation and detail coefficients according to the Mallat decomposition scheme. In each decomposition level, a part of the CO_2_ signal energy is stored in approximation coefficients, representing the CO_2_ signal trend and the rest of the energy is kept in the detail coefficients. In this way, part of the signal energy is suppressed in the detail coefficients. Regarding the decomposition level, we use the fact that the higher the decomposition level, the more energy is removed from the CO_2_ trend and the resulting signal morphology is more distorted. We experimentally set the minimal energy which should be stored in the signal trend as 75% (n = 10), minimal CO_2_ signal change 5% (n = 6) from the original CO_2_ signal, and their average (n = 6). These decomposition settings are used for the comparative analysis of the best wavelet settings for improvement of the CO_2_ prediction accuracy.

As the input signals, we use one-day and week signals. Each of these CO_2_ signals was predicted by using one of the twelve different settings of the ANN network, differing in the number of neurons within their hidden layers. This testing also points out suitable settings of the ANN.

**Figure 6:**
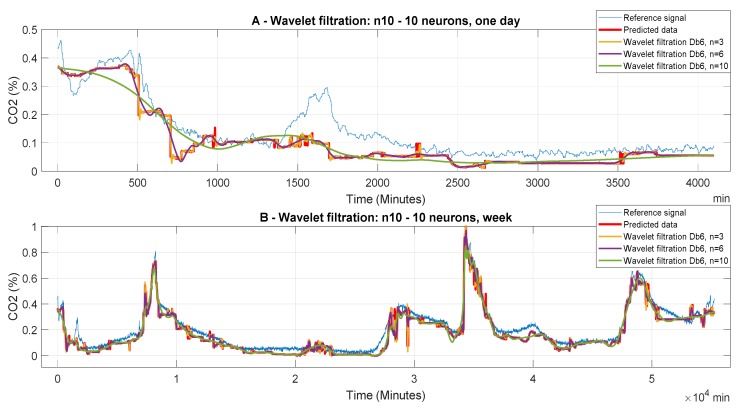
CO_2_ concentration for different time period using model 1 (n10 ot 10 neurons). Wavelet-based filtration is performed for Db6 with level of decomposition, n = 3, 6, and 10.

**Figure 7:**
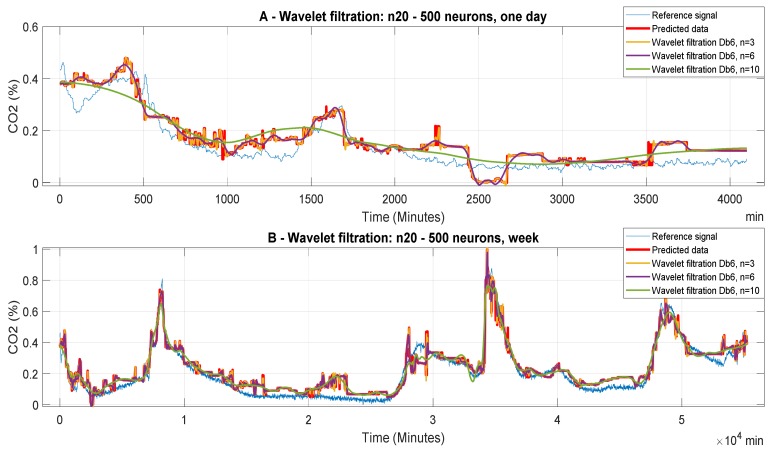
CO_2_ concentration for different time periods using Model 12 (n20 to 500 neurons). Wavelet-based filtration is performed for Db6 with level of decomposition, n = 3, 6, and 10.

**Figure 8:**
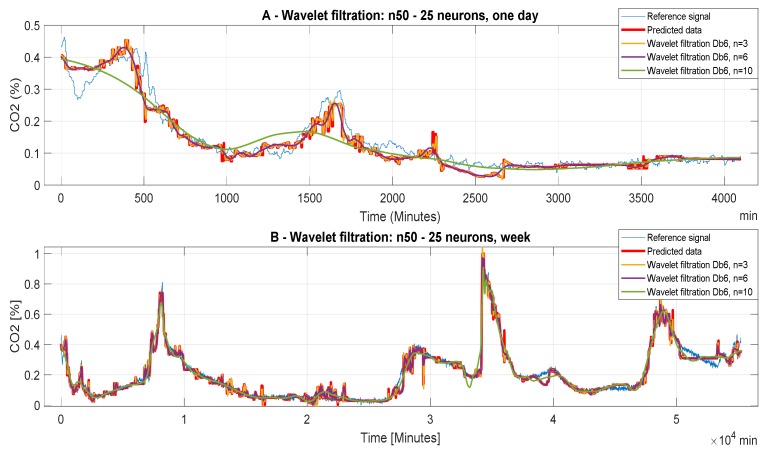
CO_2_ concentration for different time periods using Model 3 (n50 to 25 neurons). Wavelet-based filtration is performed for Db6 with level of decomposition, n = 3, 6, and 10. Concentration for different time periods using ANN model 12.

The following Figure 6, Figure 7 and Figure 8 report CO_2_ concentrations acquired and predicted within different time periods. Signals are processed by using all the levels of the decomposition to evaluate the effect of the decomposition on the wavelet-based smoothing. Figure 6, Figure 7 and Figure 8 report application of the mother’s wavelet Db6 (Daubechies). All of the signals are compared with the reference signal.

Figure 6 represents the filtered signals which were processed by the ANN, containing 10 neurons in the first hidden layer and 10 neurons in the second layer; it compares the predicted and the reference signals. In the first case (Figure 6A), it is one-day data. It is apparent that n = 10 is not suitable for the wavelet settings due to significant distortion of the filtered signal as comparing with the reference signal. Thus, there is high suppression of the CO_2_ information. The graph shows that the signal predicted by the ANN and the consequent filtered signals in all cases of wavelet filtration show a considerable deviation as compared with the reference signal. In the second case (Figure 6B), we report a week prediction. In this case, we observe, for all variants of wavelet filtration, a relatively accurate prediction of the CO_2_ concentration as compared with the reference signal, taking into account the purpose of the predictions. A more accurate assessment of the quality of prediction and subsequent filtration is performed using MSE analysis and correlation analysis.

**Figure 9:**
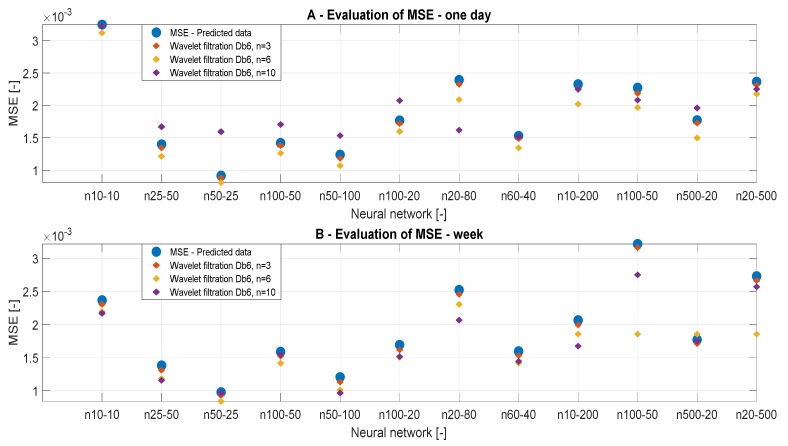
MSE values, a comparison of reference signal with the wavelet-based filtration signals.

**Figure 10:**
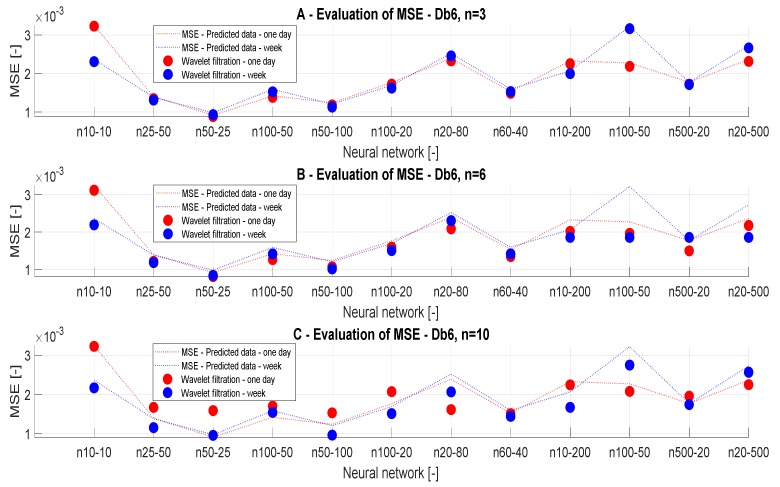
MSE values, a comparison of the reference signal with different wavelet settings.

Figure 7 represents filtered signals, predicted with the ANN with 20 neurons in the first hidden layer and 500 neurons in the second layer (model 12). In this particular case, we can observe a more accurate prediction against the reference signal. In the first case (Figure 7A), which belongs to analyzed daily signals, it is possible to observe the generated parasitic data for all possibilities of wavelet filtration. Again, the predicted data and the wavelet filtration data are inaccurate with respect to the reference signal. Their next evaluation will be given by objective evaluation. We can also note large distortions and loss of important information when n = 10. In the second case (Figure 7B), which is related to the predicted weekly data, we receive the most accurate prediction regarding the reference. By comparing the prediction signal in Figure 9 and Figure 10, it becomes apparent that the settings of the ANN have a significant impact on the prediction accuracy

Figure 8 refers to the ANN with 50 neurons in the first hidden layer and 25 neurons in the second layer (Model 3). Here, in each (Figure 8A,B) setting the decomposition level n = 10 is the worst, but in (Figure 8A) important information is suppressed in the analyzed data and information are lost in (Figure 8B), this signal distortion is not very large and for the purpose of predicting the CO_2_ concentration this decomposition level is satisfactory. For the data where the decomposition levels n = 3 and 6 has been used, it is not possible to determine with precision which of the decomposition levels are more acceptable, MSE and correlation analysis should decide again. Within the testing of the wavelet-based settings, it was observed that the neuron’s settings of ANN have a significant impact on the accuracy of the CO_2_ prediction. In this context, we can note that the ANN Model 3 has the best accuracy of the wavelet-based filtration. Testing has shown that the use of weekly data is the most appropriate for the intended purposes of CO_2_ concentration prediction. The next important finding was that the level of decomposition n = 10 is suitable for the weekly CO_2_ prediction data, but unsuitable for daily data due to excessive suppression of the signal details, and thus bad overall prediction. These findings are only preliminary results. Objective findings should be, consequently, done by using analysis based on the MSE and correlation coefficient.

#### 3.3.3. MSE Analysis

In this section, we present the results of the MSE analysis. Figure 9 and Figure 10 represent MSE results for the filtration with Db6, n = 3, n = 6, and n = 10 for individual predicted CO_2_ signals. The MSE values should converge to zero in each case. We present the MSE values in the dependence of the time range of the CO_2_ measurements, but also on the ANN settings and the wavelet settings. Table 4 and Table 5 summarize the MSE values. MSE should primarily verify optimal settings of the decomposition level. In each case of MSE, we compared the results with the reference signal.

Table 4 and Table 5 presents the MSE values in the dependence on the setting of ANN. In Table 4, we present the results of the one-day data. According to the analysis, we note that the level of the decomposition n = 6 is most accurate. In the case of n = 10 (Table 4), we achieved the greatest values of MSE. Thus, we can state that this level of decomposition is unsuitable. In Table 5, we present MSE values for weekly data. In this case, we achieved the best MSE values for n = 10. MSE values for n = 3 (Table 5) are most different from the reference signal. The ANN models 10 and 12 showed the best MSE values for n = 6 (Table 5). By comparing values of MSE analysis for the ANN Model 3 of a one-day signal (Table 4), we get a clear conclusion about the influence of the decomposition level. The principle of the MSE analysis works on the principle than lower values indicate better results, and therefore the decomposition level n = 6 is the most suitable based on the real results. From the MSE analysis, we can conclude that weekly data are not affected by the decomposition level used and it is appropriate to use the decomposition level around n = 6 to 10 for these data, whereas for daily signals it is suitable to use the decomposition level n = 3 to 6.

Figure 10 presents the same results, but they are classified by type of wavelet. On the basis of the MSE analysis, we found that the best alternative for CO_2_ settings is using weekly predicted data, using the ANN Model 3 and settings of the decomposition level n = 6 to 10, because these values represent a compromise regarding the decomposition level and settings of the ANN.

#### 3.3.4. Correlation Analysis

**Figure 11:**
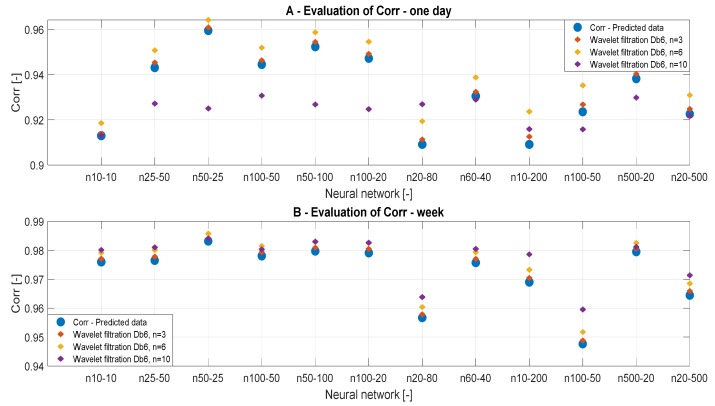
Correlation analysis of the reference signal and individual wavelet settings for two time periods: (**A**) one day and (**B**) week.

**Figure 12:**
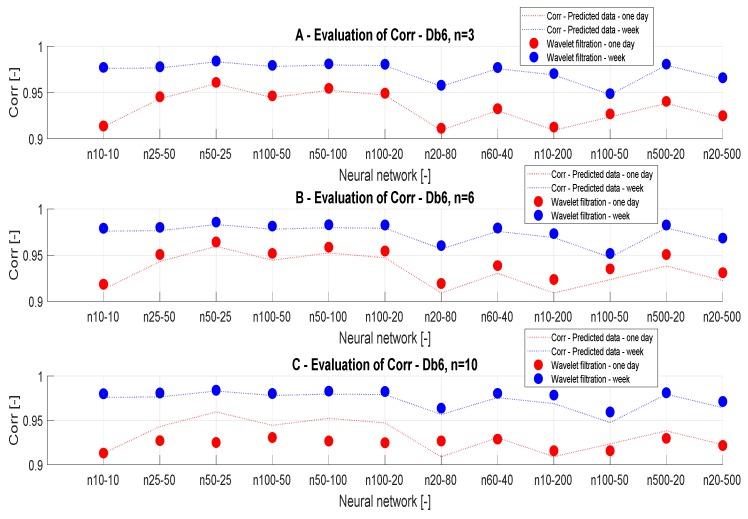
Correlation results, a comparison of the reference signal with wavelet-based filtration results.

To confirm the results of the MSE analysis, we also used the correlation analysis, investigating the linear dependency between the reference signals and the filtered signals. Table 6 and Table 7 summarize the correlation results for different wavelets, time periods, and the number of neurons. Figure 11 and Figure 12 bring the graphical trend representation of the correlation coefficient for different wavelet settings.

On the basis of the analysis from Table 4 and Figure 11, we can state that the decomposition level has a greater impact on the analysis of daily data. The results of the correlation analysis are the same as the result of the MSE analysis and it can be stated that the conclusions were correct. On the basis of the correlation analysis, we can confirm the conclusion that the decomposition level n = 10 causes a significant signal distortion in daily data. This phenomenon is especially noticeable in Figure 11A. In contrast, in the analysis of weekly data in Figure 11B, the decomposition level n = 10 appears to be the best. This could be caused by the type of the signal, particularly its length. Figure 12 presents the correlation analysis results which are classified by the filtration method used.

#### 3.3.5. Analysis of Wavelet Selection for CO_2_ Prediction

In this section, we evaluate the influence of the selected wavelet on the quality of the predicted signal. As we stated before, we analyze the Db6 wavelet from the family Daubechies, coif1 from the family Coiflets, and sym1 from the family Symlets, when the level of the decomposition and filter settings were the same for all of the cases. This testing should bring an answer to the question of the influence of characteristic wavelet features on the predicted CO_2_ signal. We only work with the n = 3 since the individual wavelet types must be compared.

**Figure 13:**
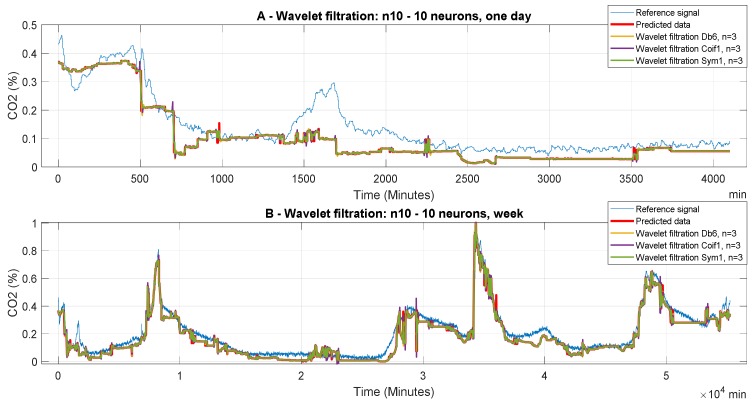
Dependence of CO_2_ prediction on the time period for the ANN Model 1.

Figure 13 represents the information about the output filtered signal with the use of different wavelets. In this case, it is relatively apparent that the selection of different wavelets does not have a significant impact on the filtered results for all of the time periods. In addition, it is obvious that the ANN Model 1 does not give satisfactory results for CO_2_ prediction due to an insufficient similarity of the reference signals with the predicted results. This phenomenon is mainly observable in the case of daily data. This fact causes signal loss and distortion.

**Figure 14:**
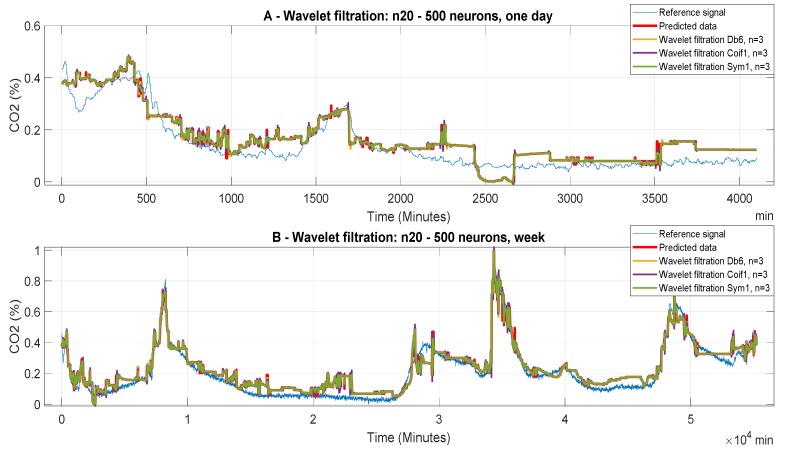
The dependency of CO_2_ concentration on the time period for the neural network model 12 (n20 to 500).

**Figure 15:**
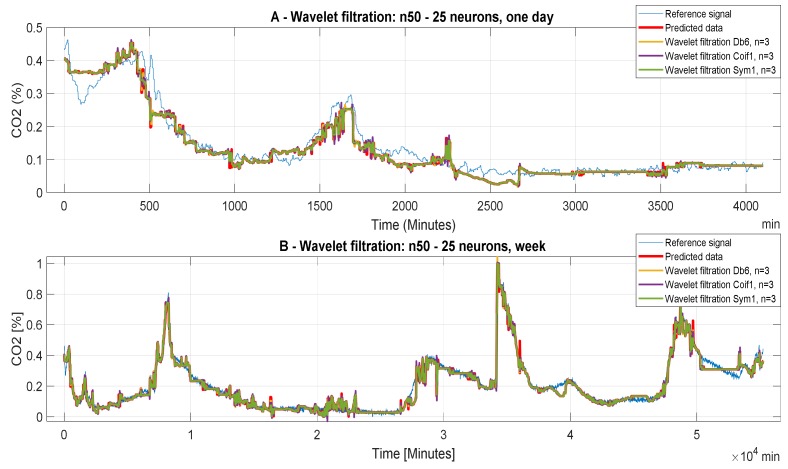
The dependency of CO_2_ concentration on the time period for the neural network with 400 neurons.

Figure 14 and Figure 15 also present the information that individual wavelets do not bring significantly comparable information. These graphical representations confirm previous conclusions about the settings of ANN regarding the preciseness of the CO_2_ prediction.

#### 3.3.6. MSE Analysis for Different Wavelets

**Figure 16:**
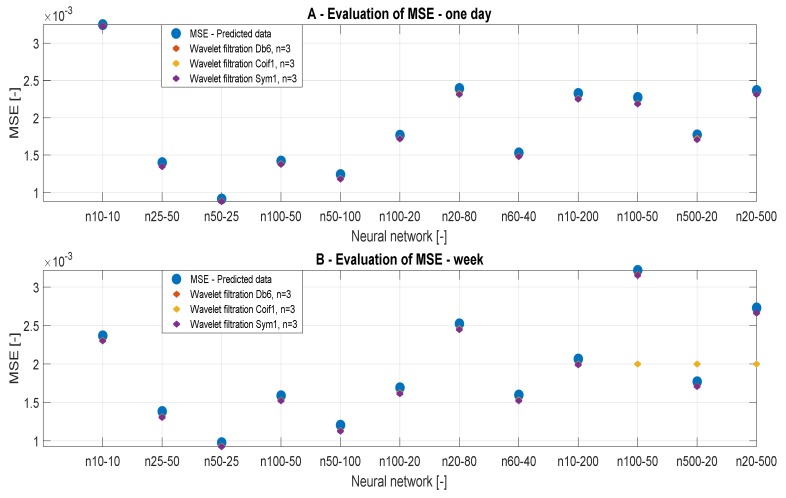
MSE values, a comparison of the reference signals with the wavelet-based filtration.

**Figure 17:**
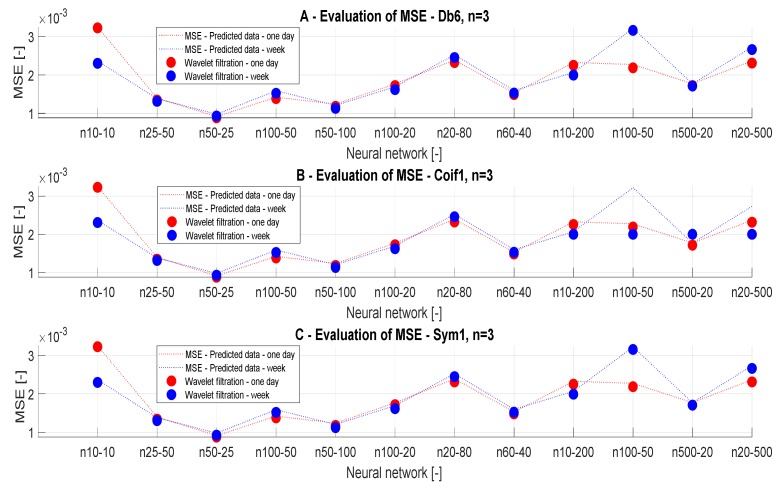
Trend characteristics for MSE values, a comparison of the reference signals with the wavelet-based filtration for different neural network settings and time prediction.

In Table 8 and Table 9, we present the MSE analysis for different wavelets (Db6, Coif1, and Sym), for various time intervals and the ANN settings. The graphical distribution of these results is presented in Figure 16 and Figure 17. According to these results, we obtain similar results for these wavelets. These wavelets were selected based on the testing among other wavelets. These wavelets achieved the best results as in the MSE analysis, and also in the correlation analysis.

When we use the definition of MSE, it is obvious that MSE differences are insignificant, and it is not possible to objectively classify which wavelet is the most suitable for the analysis. Similar results are also achieved in the case of the weekly predictions.

Figure 17 presents the trend evaluation of MSE values for different wavelets with the level of decomposition n = 3 depending on the ANN configuration.

For the one-day prediction, we achieved that MSE results are comparable for all the wavelets. As well as for longer predictions, we obtain the results which do not exhibit significant differences. Figure 17 also presents MSE analysis results. In this case, we compare trend evaluation for each wavelet on the ANN settings and the prediction periods. These results show a monotonic tendency of the prediction accuracy depending on the number of neurons and the prediction time. In most cases, it applies the stated conclusion, only for ANN models 4, 11, and 12, we see specific values for wavelet filtration using wavelet coif1.

#### 3.3.7. Correlation Analysis for Different Wavelets

**Figure 18:**
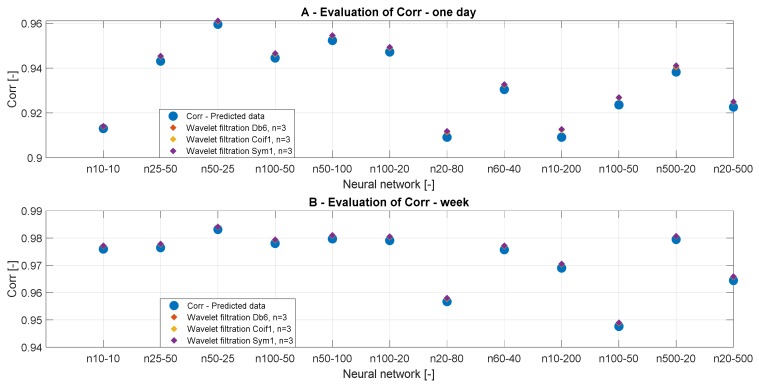
Analysis of correlation values, a comparison for different wavelets depending on various time periods and the neural network settings.

**Figure 19:**
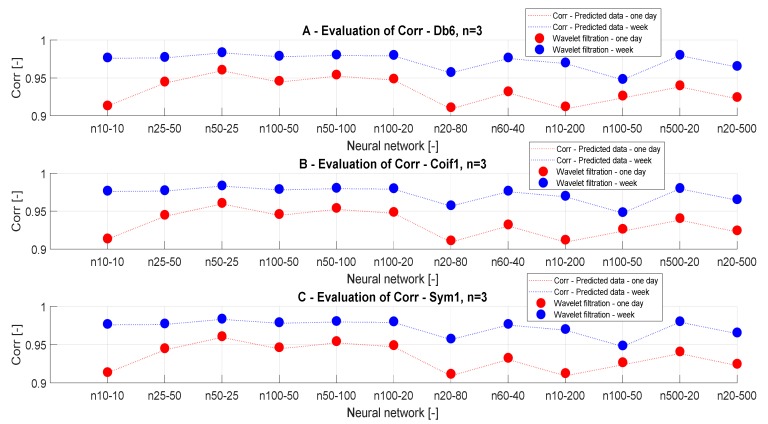
Analysis of correlation values, a comparison of individual wavelet settings for each time period of prediction and the number of neurons.

In this section, we present the results of the correlation analysis for different wavelets. This analysis should confirm the results of the MSE analysis. Figure 18 and Figure 19 show (Table 10 and Table 11) the trend correlation characteristics for different wavelets with the level of the decomposition n = 3, depending on the ANN settings and the time interval of prediction. In Figure 18B, we have a comparison of the correlation analysis for weekly data. As we present the correlation results, it is obvious that differences are negligible. These results prove that the selection of the mother’s wavelet for the wavelet-based prediction does not have a substantial impact on the prediction accuracy.

## 4. Discussion

In this paper, we report the comparative analysis of various wavelet settings with the goal of improving the CO_2_ signal prediction. The predicted results from the ANN standard contain glitches and artifacts which more or less deteriorate the quality of the CO_2_ signal accuracy. Therefore, we analyze the hybrid system that consists of the ANN prediction, with the consequent filtration procedure, based on the wavelet transformation.

In the wavelet analysis, we are mainly focused on the wavelet decomposition level (n = 3, 6, and 10) and different mother’s wavelets, including Daubechies (db6’s), Coiflets (coif1’e), and Symlet (sym1). We test these various wavelet settings for ten modifications of the ANN architecture with the goal of evaluating the best wavelet settings and the most suitable ANN settings for the CO_2_ signal prediction.

The experimental testing is done for the one-day and one-week data of the CO_2_ concentration. All the evaluations are performed objectively, where we compare the predicted results from the ANN and wavelet filtration against the reference CO_2_ signals. Such a procedure objectively evaluates the quality and robustness of each wavelet setting for the CO_2_ signal prediction optimization. 

In this objective analysis, we use the following two metrics: (1) MSE, which is a type of the error function, expressing a difference between the reference signal and prediction and (2) LC, expressing the linear dependence level between the reference signal and prediction. On the basis of the MSE analysis, we conclude n = 6 as the most accurate for the CO_2_ prediction as compared with other decompositions. Regarding the time period of the CO_2_ signal, we found the week prediction to be the most accurate with n = 6 and 10. Consequently, we verify the MSE results by the correlation analysis. On the basis of the correlation analysis, we confirm the conclusion that the decomposition level n = 10 causes a significant signal distortion in the daily data. Furthermore, n = 6 appears as the best compromise of wavelet settings for the CO_2_ prediction in the case of one-day data. Contrarily, in the case of the week-data, we noticed n = 10 as the best compromise.

On the one hand, the experimental settings and testing show the tendency and potential of the wavelet-based filtration for the optimization of CO_2_ prediction accuracy. On the other hand, there are still open research questions regarding wavelet applications. Mainly, the potential of this system could be optimized by incorporating the methods of artificial intelligence with the goal of the autonomous selection of the most appropriate settings for the wavelet-based filtration.

## 5. Conclusions

This article proposed the implementation of a new method to determine the occupancy of monitored areas in IB by predicting the course of CO_2_ concentration from the measured indoor temperature and indoor relative humidity. The article introduced the procedure of programming KNX modules using the ETS 5 SW tool to simulate the control of operational and technical functions in SH. Additionally, KNX-IoT connectivity was implemented to store and, subsequently, process the measured data using ANN (MLP).

A crucial part of the proposed method was to increase the accuracy of CO_2_ predictions by wavelet transformation by suppressing the additive noise from the predicted signal. In this article, we present the comparative analysis of different settings of the wavelet transformation for the CO_2_ signal prediction. We mainly pay attention to the effect of the level decomposition and type of mother’s wavelet on the prediction accuracy. We experimentally set these settings and, consequently, evaluated settings impact on the prediction accuracy. In selected experiments, the accuracy of the prediction was better than 98%. On the basis of the above results and documented experiments, it can be stated that the main goal of the authors, "finding the optimal setting of individual parameters of the wavelet transform in the additive noise suppression application with an emphasis on increasing the accuracy of predictions of CO_2_ concentration from measured values of indoor temperature and indoor relative humidity" were unambiguously met.

Future trends in our analysis could be aimed at building the optimization scheme based on artificial intelligence employing either genetic algorithms or methods of evolutionary computing with the goal of optimal selection of wavelet settings. Such optimization could bring new possibilities of CO_2_ modeling, and autonomous classification of the wavelet settings for particular CO_2_ signals.

Future work could deal with IB automation in the context of optimized management of operational-technical functions to reduce operating costs, increase control and comfort (for example HVAC control, light control, and blinds control) with different technological systems (for example KNX RF technology, Bacnet technology, Lonworks technology, and Loxone technology) and platforms (for example IoT, SH, SHC, and SC) based on occupancy recognition of humans in IB spaces and indoor monitoring of human positioning. Our objectives are optimization and practical implementation of a novel design method to monitor human daily living activities in the SH, indirect methods for human presence monitoring in the IB, and lifelong learning of occupant behavior in SH systems within the ethical and privacy-preservation approach to SH.

## Figures and Tables

**Table 1:** Prediction results using an interval of May 2 at 10:08:06 and ended on the May 10 (based on validation partition).

Model Number	Number of Neurons		Accuracy (%)	LC	MSE
	Layer 1	Layer 2			
1	10	10	95.2	0.976	2.368 × 10^−3^
2	25	50	95.4	0.976	1.383 × 10^−3^
3	50	25	96.7	0.983	9.78 × 10^−3^
4	100	50	95.7	0.978	1.589 × 10^−3^
5	50	100	96.0	0.979	1.205 × 10^−3^
6	100	20	95.9	0.979	1.693 × 10^−3^
7	20	80	91.6	0.956	2.525 × 10^−3^
8	60	40	95.2	0.975	1.597 × 10^−3^
9	10	200	94.0	0.969	2.067 × 10^−3^
10	100	500	90.0	0.947	3.22 × 10^−3^
11	500	20	95.9	0.979	1.77 × 10^−3^
12	20	500	93.1	0.964	2.73 × 10^−3^

**Table 2:** Wavelet filtration settings for CO_2_ signal smoothing.

Threshold	Wavelet Filter Thresholding	Thresholding Rescaling
rigrsure	soft thresholding	for no rescaling

**Table 3:** Wavelet functions for analysis of CO_2_ prediction.

**Daubechies**	‘Db6’s
**Coiflets**	‘coif1’e
**Symlets**	‘sym1’

**Table 4:** Mean square error (MSE) values for wavelet-based filtration of CO_2_ signals (one day).

Model Number	Number of Neurons		Day			
	Layer 1	Layer 2	ref	n = 3	n = 6	n = 10
1	10	10	3.241 × 10^−3^	3.231 × 10^−3^	3.122 × 10^−3^	3.229 × 10^−3^
2	25	50	1.399 × 10^−3^	1.346 × 10^−3^	1.216 × 10^−3^	1.671 × 10^−3^
3	50	25	9.132 × 10^−4^	8.826 × 10^−4^	8.087 × 10^−4^	1.593 × 10^−3^
4	100	50	1.421 × 10^−3^	1.382 × 10^−3^	1.264 × 10^−3^	1.707 × 10^−3^
5	50	100	1.240 × 10^−3^	1.183 × 10^−3^	1.069 × 10^−3^	1.535 × 10^−3^
6	100	20	1.767 × 10^−3^	1.722 × 10^−3^	1.597 × 10^−3^	2.075 × 10^−3^
7	20	80	2.393 × 10^−3^	2.326 × 10^−3^	2.089 × 10^−3^	1.618 × 10^−3^
8	60	40	1.531 × 10^−3^	1.487 × 10^−3^	1.344 × 10^−3^	1.516 × 10^−3^
9	10	200	2.328 × 10^−3^	2.254 × 10^−3^	2.021 × 10^−3^	2.249 × 10^−3^
10	100	50	2.274 × 10^−3^	2.188 × 10^−3^	1.968 × 10^−3^	2.082 × 10^−3^
11	500	20	1.773 × 10^−3^	1.726 × 10^−3^	1.497 × 10^−3^	1.960 × 10^−3^
12	20	500	2.367 × 10^−3^	2.315 × 10^−3^	2.177 × 10^−3^	2.253 × 10^−3^

**Table 5:** MSE values for wavelet-based filtration of CO_2_ signals (week).

Model Number	Number of Neurons		Week			
	Layer 1	Layer 2	ref	n = 3	n = 6	n = 10
1	10	10	2.368 × 10^−3^	2.307 × 10^−3^	2.195 × 10^−3^	2.172 × 10^−3^
2	25	50	1.383 × 10^−3^	1.310 × 10^−3^	1.182 × 10^−3^	1.156 × 10^−3^
3	50	25	9.780 × 10^−4^	9.295 × 10^−4^	8.382 × 10^−4^	9.618 × 10^−4^
4	100	50	1.589 × 10^−3^	1.524 × 10^−3^	1.415 × 10^−3^	1.545 × 10^−3^
5	50	100	1.205 × 10^−3^	1.129 × 10^−3^	1.013 × 10^−3^	9.649 × 10^−4^
6	100	20	1.693 × 10^−3^	1.619 × 10^−3^	1.504 × 10^−3^	1.516 × 10^−3^
7	20	80	2.525 × 10^−3^	2.457 × 10^−3^	2.306 × 10^−3^	2.067 × 10^−3^
8	60	40	1.597 × 10^−3^	1.530 × 10^−3^	1.417 × 10^−3^	1.444 × 10^−3^
9	10	200	2.067 × 10^−3^	1.996 × 10^−3^	1.857 × 10^−3^	1.675 × 10^−3^
10	100	50	3.220 × 10^−3^	3.165 × 10^−3^	1.857 × 10^−3^	2.753 × 10^−3^
11	500	20	1.770 × 10^−3^	1.712 × 10^−3^	1.857 × 10^−3^	1.746 × 10^−3^
12	20	500	2.730 × 10^−3^	2.667 × 10^−3^	1.857 × 10^−3^	2.569 × 10^−3^

**Table 6:** Correlation analysis for the reference signals and wavelet-based filtration (day).

Model Number	Number of Neurons		Day			
	Layer 1	Layer 2	ref	n = 3	n = 6	n = 10
1	10	10	0.913	0.914	0.919	0.913
2	25	50	0.943	0.945	0.951	0.927
3	50	25	0.960	0.961	0.964	0.925
4	100	50	0.945	0.946	0.952	0.931
5	50	100	0.952	0.955	0.959	0.927
6	100	20	0.947	0.949	0.955	0.925
7	20	80	0.909	0.911	0.919	0.927
8	60	40	0.931	0.932	0.939	0.929
9	10	200	0.909	0.913	0.924	0.916
10	100	50	0.924	0.927	0.935	0.916
11	500	20	0.938	0.940	0.951	0.930
12	20	500	0.923	0.925	0.931	0.922

**Table 7:** Correlation analysis for the reference signals and wavelet-based filtration (week).

Model Number	Number of Neurons		Week			
	Layer 1	Layer 2	ref	n = 3	n = 6	n = 10
1	10	10	0.976	0.977	0.979	0.980
2	25	50	0.977	0.978	0.980	0.981
3	50	25	0.983	0.984	0.986	0.984
4	100	50	0.978	0.979	0.982	0.980
5	50	100	0.980	0.981	0.983	0.983
6	100	20	0.979	0.981	0.983	0.983
7	20	80	0.957	0.958	0.960	0.964
8	60	40	0.976	0.977	0.979	0.980
9	10	200	0.969	0.970	0.973	0.979
10	100	50	0.947	0.949	0.952	0.960
11	500	20	0.980	0.981	0.983	0.981
12	20	500	0.965	0.966	0.969	0.971

**Table 8:** MSE values for the reference signals and wavelet-based filtered signals.

Model Number	Number of Neurons		Day			
	Layer 1	Layer 2	ref	n = 3	n = 6	n = 10
1	10	10	3.249 × 10^−3^	3.231 × 10^−3^	3.227 × 10^−3^	3.224 × 10^−3^
2	25	50	1.399 × 10^−3^	1.346 × 10^−3^	1.349 × 10^−3^	1.345 × 10^−3^
3	50	25	9.132 × 10^−4^	8.826 × 10^−4^	8.803 × 10^−4^	8.776 × 10^−4^
4	100	50	1.421 × 10^−3^	1.382 × 10^−3^	1.383 × 10^−3^	1.376 × 10^−3^
5	50	100	1.240 × 10^−3^	1.183 × 10^−3^	1.185 × 10^−3^	1.179 × 10^−3^
6	100	20	1.767 × 10^−3^	1.722 × 10^−3^	1.724 × 10^−3^	1.716 × 10^−3^
7	20	80	2.393 × 10^−3^	2.326 × 10^−3^	2.322 × 10^−3^	2.313 × 10^−3^
8	60	40	1.530 × 10^−3^	1.487 × 10^−3^	1.484 × 10^−3^	1.479 × 10^−3^
9	10	200	2.328 × 10^−3^	2.254 × 10^−3^	2.259 × 10^−3^	2.251 × 10^−3^
10	100	50	2.274 × 10^−3^	2.188 × 10^−3^	2.192 × 10^−3^	2.186 × 10^−3^
11	500	20	1.773 × 10^−3^	1.726 × 10^−3^	1.716 × 10^−3^	1.708 × 10^−3^
12	20	500	2.367 × 10^−3^	2.315 × 10^−3^	2.317 × 10^−3^	2.313 × 10^−3^

**Table 9:** MSE values for the reference signals and wavelet-based filtered signals.

Model Number	Number of Neurons		Week			
	Layer 1	Layer 2	ref	n = 3	n = 6	n = 10
1	10	10	2.368 × 10^−3^	2.307 × 10^−3^	2.306 × 10^−3^	2.300 × 10^−3^
2	25	50	1.383 × 10^−3^	1.310 × 10^−3^	1.315 × 10^−3^	1.307 × 10^−3^
3	50	25	9.780 × 10^−4^	9.295 × 10^−4^	9.308 × 10^−4^	9.256 × 10^−4^
4	100	50	1.590 × 10^−3^	1.524 × 10^−3^	1.527 × 10^−3^	1.521 × 10^−3^
5	50	100	1.205 × 10^−3^	1.129 × 10^−3^	1.133 × 10^−3^	1.125 × 10^−3^
6	100	20	1.693 × 10^−3^	1.619 × 10^−3^	1.625 × 10^−3^	1.615 × 10^−3^
7	20	80	2.525 × 10^−3^	2.457 × 10^−3^	2.458 × 10^−3^	2.448 × 10^−3^
8	60	40	1.597 × 10^−3^	1.530 × 10^−3^	1.531 × 10^−3^	1.522 × 10^−3^
9	10	200	2.067 × 10^−3^	1.996 × 10^−3^	1.999 × 10^−3^	1.991 × 10^−3^
10	100	50	3.220 × 10^−3^	3.165 × 10^−3^	1.999 × 10^−3^	3.152 × 10^−3^
11	500	20	1.770 × 10^−3^	1.712 × 10^−3^	1.999 × 10^−3^	1.708 × 10^−3^
12	20	500	2.730 × 10^−3^	2.667 × 10^−3^	1.999 × 10^−3^	2.664 × 10^−3^

**Table 10:** Correlation analysis for the reference signals and wavelet-based filtration.

Model Number	Number of Neurons		Day			
	Layer 1	Layer 2	ref	n= 3	n= 6	n= 10
1	10	10	0.913	0.914	0.914	0.914
2	25	50	0.943	0.945	0.945	0.945
3	50	25	0.960	0.961	0.961	0.961
4	100	50	0.945	0.946	0.946	0.947
5	50	100	0.952	0.955	0.954	0.955
6	100	20	0.947	0.949	0.949	0.949
7	20	80	0.909	0.911	0.912	0.912
8	60	40	0.931	0.932	0.933	0.933
9	10	200	0.909	0.913	0.912	0.913
10	100	50	0.924	0.927	0.927	0.927
11	500	20	0.938	0.940	0.941	0.941
12	20	500	0.923	0.925	0.925	0.925

**Table 11:** Correlation analysis for the reference signals and wavelet-based filtration.

Model Number	Number of Neurons		Week			
	Layer 1	Layer 2	ref	n= 3	n= 6	n= 10
1	10	10	0.976	0.977	0.977	0.977
2	25	50	0.977	0.978	0.978	0.978
3	50	25	0.983	0.984	0.984	0.984
4	100	50	0.978	0.980	0.979	0.979
5	50	100	0.980	0.981	0.981	0.981
6	100	20	0.979	0.981	0.980	0.981
7	20	80	0.957	0.958	0.958	0.958
8	60	40	0.976	0.977	0.977	0.977
9	10	200	0.969	0.970	0.970	0.971
10	100	50	0.948	0.949	0.949	0.949
11	500	20	0.980	0.981	0.980	0.981
12	20	500	0.965	0.966	0.966	0.966

## Abbreviations

The following abbreviations are used in this manuscript:

ANN	Artificial neural network
MLP	Multilayer perceptron
CC	Cloud computing
IoT	Internet of Things
SH	Smart home
KNX	Konnex (standard EN 50090, ISO/IEC 14543)
MQTT	Message queuing telemetry transport
ETS	Engineering tool software
SW	Software
CO_2_	Carbon dioxide
CoAP	Constrained application protocol
XMPP	Extensible messaging and presence protocol
IBM SPSS	Statistical package for the social sciences from the company IBM
LMA	Levenberg–Marquardt algorithm
BRM	Bayesian regulation method
ppm	Parts per million
LMS	Least mean square
AF	Adaptive filtration
SCADA	Supervisory Control and Data Acquisition
SCG	Scaled conjugate gradient
DWT	Discrete wavelet transformation
STFT	Short-time Fourier transformation
TP	Twisted pair (also known as KNX bus)
PL	Powerline
RF	Radio frequency
IP	Industrial protocol, ethernet
MSE	Mean square error
LC	Linear correlation
EB312	Laboratory room at the VSB TU Ostrava
FEI	Faculty of Electrical Engineering and Computer Science
VSB	TU Ostrava - VSB - Technical University of Ostrava
db6’s	Daubechies
coif1’e	Coiflets
sym1	Symlet
HVAC	Heating, ventilation, and air conditioning
